# Player-Level Tackle Training Interventions in Tackle-Collision Sports: A Systematic Scoping Review

**DOI:** 10.1186/s40798-025-00888-9

**Published:** 2025-09-10

**Authors:** Demi Davidow, Lara Paul, Ben Jones, Ameer Hohlfeld, Seipati Rasenyalo, Kathryn Dane, Isla J. Shill, Sharief Hendricks

**Affiliations:** 1 Division of Physiological Sciences, Department of Human Biology, Faculty of Health Sciences, University of Cape Town, Newlands, Cape Town, 7725 South Africa; 2 Health Through Physical Activity, Lifestyle and Sport Research Centre, Department of Human Biology, Faculty of Health Sciences, University of Cape Town, Newlands, Cape Town, 7725 South Africa; 3 South African Medical Research Council, Cochrane South Africa, South Africa; 4 Division of Epidemiology and Biostatistics, School of Public Health, Faculty of Health Sciences, University of Cape Town, Observatory, Cape Town 7925, South Africa; 5 Carnegie Applied Rugby Research (CARR) Centre, Carnegie School of Sport, Leeds Beckett University, Leeds, UK; 6 School of Behavioural and Health Sciences, Faculty of Health Sciences, Australian Catholic University, Brisbane, QLD Australia; 7 England Performance Unit, Rugby Football League, Manchester, UK; 8 Premiership Rugby, London, UK; 9 Discipline of Physiotherapy, School of Medicine, Trinity College Dublin, Dublin, Ireland; 10 Sport Injury Prevention Research Centre, Faculty of Kinesiology, University of Calgary, Calgary, AB Canada; 11 Hotchkiss Brain Institute, University of Calgary, Calgary, AB Canada

**Keywords:** Training, Performance, Prevention, Rugby, American Football

## Abstract

**Background:**

In tackle-collision sports, the tackle has the highest incidence, severity, and burden of injury. Head injuries and concussions during the tackle are a major concern within tackle-collision sports. To reduce concussion and head impact risk, evaluating optimal tackle techniques to inform tackle-related prevention strategies has been recommended. The purpose of this study was to perform a systematic scoping review of player-level tackle training intervention studies in all tackle-collision sports.

**Methods:**

The Arksey and O’Malley’s five-stage scoping review process and Levac et al.’s framework were used, along with the Preferred Reporting Items for Systematic Reviews and Meta-Analysis extension for Scoping Reviews (PRISMA-ScR) checklist. The main inclusion criteria were that the study included an intervention aimed at improving a player’s tackle abilities, and the intervention had to be delivered/implemented at the player-level in a training setting.

**Results:**

Thirteen studies were included in this review, seven studies in American Football (54%), followed by a combined cohort of rugby union and rugby league players (three studies; 23%), rugby union (two studies; 15%), and one study reported on a rugby league cohort (8%). Studies focused primarily on the tackler, with the intervention incorporating a form of instruction or feedback, delivered through video or an expert coach. Other interventions included an 8-week strength and power training programme, designing practice sessions based on baseline data, and helmetless training in American Football. All interventions demonstrated a favourable change in the outcome measured—which included tackler and ball-carrier kinematics based on motion capture video, tackler proficiency scoring, tackling task analysis, head impact frequencies by xPatch head-impact sensor technology, head impact kinematics using head-impact sensors (helmet or skin patches) and football tackle kinematics with motion capture systems or video.

**Conclusion:**

This review shows that a range of studies have been undertaken focusing on player-level training interventions. The quality of studies were rated as ‘good’, and all studies showed improvements in outcome measures. Coaches and policy makers should ensure tackle technique is profiled alongside other player characteristics, and an evidence-based approach to improving player tackling is adopted, improving both performance and reducing injury risk.

**Key Points:**

- Only 13 studies tested or implemented interventions at the player level in tackle-collision sports.
- The focus of the studies was primarily on the tackler, with the interventions incorporating a form of instruction or feedback, which was delivered through video or an expert coach.
- Other interventions included an 8-week strength and power training programme, designing practice sessions based on baseline data, and helmetless training in American Football.
- All interventions demonstrated a favourable change in the outcome measure and provide coaches and policymakers with tackle training insights.

**Registration:**

The systematic scoping review was prospectively registered with OSF (registration number: 10.17605/OSF.IO/V3KZC).

**Supplementary Information:**

The online version contains supplementary material available at 10.1186/s40798-025-00888-9.

## Background

The tackle contest is a key characteristic of tackle-collision sports such as rugby union, rugby league and American Football. It occurs frequently, as it is the main action for impeding the attacker’s progression toward the goal-line and scoring points [1–6]. This frequency of occurrence combined with the physical-technical nature of the tackle expose both the tackler and ball-carrier to a high risk of injury compared to other match events (for example, scrum) [7–10]. Tackler and ball-carrier injuries are also the most severe (days away from the sport) and carries a high injury burden compared to other match events [7–10]. Head injuries and concussions in particular are a major concern within tackle-collision sports [11]. To reduce concussion and head impact risk, a recent systematic review and meta-analysis of prevention strategies for sport-related concussions and head impacts (which formed part of the 6th International Consensus Conference on Concussion in Sport, 2022) recommended that evaluating optimal tackle technique is required to inform tackle-related prevention strategy [12].

Both the ball-carrier and tackler require technical, physical, and psychological proficiency to contest the tackle [13]. Deficiencies in tackling and ball-carrying technique specifically, have been shown to increase players’ risk of injury and reduce the likelihood of a positive performance outcome [14]. For example, in rugby union, Meintjes et al. (2021) showed that overall technical proficiency scores were significantly lower for tackler and ball-carrier injuries compared to injury-free controls [15]. Studies in rugby union and rugby league have also identified physical qualities such as leg power to be associated with tackling ability [16, 17]. Changes in physical and mental fatigue have also been shown to affect players' tackling abilities [18–20].

The above mentioned video analysis and conditioning research have informed tackle coach education, contact load guidelines, and tackle laws. On the passive-active injury prevention continuum, changing tackle laws is viewed closer to the ‘passive’ end of the continuum, while broad tackle coach education moves injury prevention closer to the active end. The active end requires players or any party responsible for player safety to directly and deliberately engage with the injury prevention measure [21, 22]. Thus, in the context of the tackle, the most active measure on the continuum is player-level training interventions intended to change the player’s tackle behaviours—for example, tackle technique training [21]. Player-level interventions in this case relates to the delivery of tackle specific training components or training modalities directly to the player(s). In other words, the player(s) is engaged and present during the delivery of a training intervention aimed at improving his/her tackle ability. Recognising the need for active measures to prevent tackle injury, research has been conducted to test specific player-level tackle training interventions in tackle contact sports. These studies, however, vary in design, type of training intervention and outcome measure. In view of the need to reduce concussion and head impact risk in the tackle, a synthesis of the scope of these player-level tackle training intervention studies is warranted. Furthermore, considering the growing need for policy-makers to produce more active injury prevention measures such as tackle training interventions, a review of the literature will help identify research gaps, which will in turn help direct funding allocation for future work in this area. Therefore, the purpose of this study is to perform a systematic scoping review of player-level tackle training intervention studies in tackle-collision sports.

## Methods

A scoping review was considered the most appropriate methodological approach due to the exploratory nature of the study aim. Scoping reviews are a type of review that seeks to identify the nature and extent of research evidence for a given topic, and identify gaps in the literature [23]. Through summarising and disseminating the extent, range and nature of the latest research, a scoping review of player-level tackle training interventions may drive evidence-based practice, policy and tackle training resource development.

The current scoping review was based on Arksey and O’Malley’s five-stage scoping review process, [23] and informed Levac et al.’s framework [24, 25]. The Preferred Reporting Items for Systematic Reviews and Meta-Analysis extension for Scoping Reviews (PRISMA-ScR) checklist was used as a guideline [26, 27]. This systematic scoping review was prospectively registered with Open Science Framework (OSF) (registration number: 10.17605/OSF.IO/V3KZC).

### Stage 1: Identifying the Research Questions

The following three research questions were identified for this review:

- How many tackle training interventions have been studied within the different field-based tackle-collision sports?
- What are the details in terms of study design, outcome measures and quality for each tackle training intervention study?
- What were the changes in players' tackle ability for these studies?

### Stage 2: Identifying Relevant Studies

A systematic search of four electronic databases (PubMed, EBSCOHost, Web of Science and Scopus) was conducted for all relevant articles published between January 2000 and May 30th 2024. The search strategy used a combination of keywords: ‘Collision sport’ OR ‘Contact sport’ OR ‘Impact sport’ OR ‘Rugby’ OR ‘Rugby Union’ OR ‘Rugby Sevens’ OR ‘Rugby League’ OR ‘Australian Rules Football’ OR ‘Australian football league’ (AFL) OR ‘Canadian Football’ OR ‘Gaelic Football’ OR ‘American Football’ OR ‘Gridiron’ (each sporting code was searched separately) AND ‘tackl*’ AND ‘intervention’ OR ‘exercis*’ OR ‘program*’ OR ‘condition*’ OR ‘performance’ OR ‘injury’ OR ‘prevention’ OR ‘contact’ OR ‘technique’ OR ‘skill’ OR ‘train*’. (e.g., “Rugby Union” AND “tackl*” AND “exercis*”, or “American Football” AND “tackl*” AND “exercis*”). The electronic searches for the databases are in the Supplementary Material File. In addition, hand searching and reference checking of the full text papers that met the inclusion criteria were performed to search for other relevant studies. After completing the electronic database search, duplicates were removed, and titles screened according to the PRISMA-ScR guidelines [26, 27].

### Stage 3: Study Selection

The eligibility criteria for this review were as follows:

Inclusion criteria:

- An original research study published in a peer-reviewed journal.
- The study was published in the English language.
- The study participant cohort was tackle-collision sport athletes.
- The study included an intervention aimed at improving the tackle ability of the player i.e. the outcome measures related to changes in the player's behaviours, for the tackler and/or ball-carrier.
- The intervention was tackle-specific and delivered/implemented at the player-level in a training setting.
- The delivery of the intervention was described.

Exclusion criteria:

- Studies that included policy-level and coach-level interventions.
- Literature reviews, theses, and conference proceedings.

These eligibility criteria were selected to support and help best answer the research questions.

**Fig. 1:**
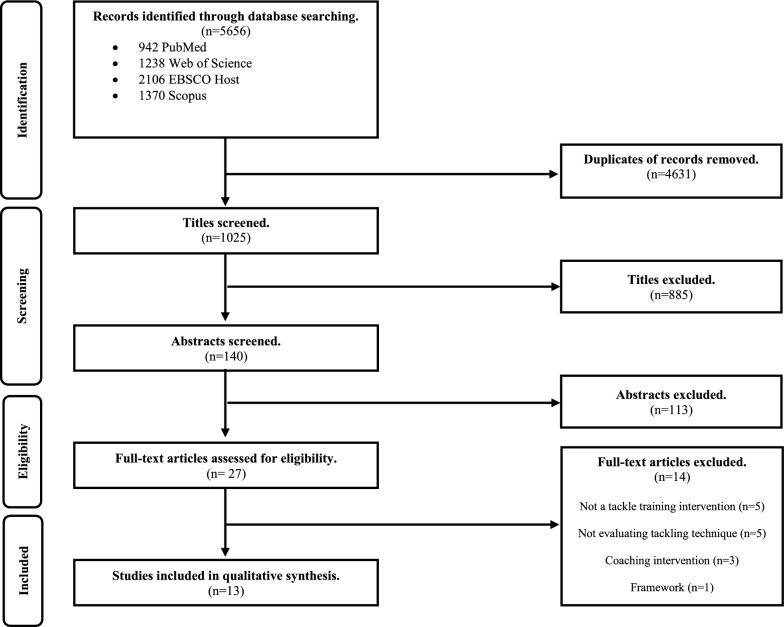
PRISMA-ScR flow diagram of literature search

Authors DD and LP screened the publications for eligibility at the title, abstract and full text level (Fig. 1). Any disagreements on article eligibility were resolved through discussions between the two reviewers, without the need for the involvement of a third author. Publications that did not meet the inclusion criteria were removed.

### Stage 4: Charting the Data

Data charting included extracting key information relevant to answering the research questions. DD conducted the data extraction, which was corroborated by SH. The following data were recorded and extracted onto an Excel spreadsheet and then presented in table format. Table 1 (categorised by sport type) charted the general characteristics of each study (author(s), year of publication, sample size, participant sex, level of participation, role in tackle), aim of study, study design, intervention type, comparator, intervention description, outcome measures and relevant findings.

**Table 1:** Shows a summary of the thirteen included studies based on participant sport cohort

Study	Sample size, gender, level of participation	Role in tackle	Aim of study	Study design	Intervention type	Comparator	Intervention description	Outcome measures	Relevant findings
*Rugby union*									
Kerr et al. (2018) [39]	50 participants - 28 male - 22 female Youth and amateur level	Tackler	Assess the effect of an educational video intervention on tackle biomechanics, in male and female players, and assess playing level response to video and repetition	Exploratory	Educational video	Player level and gender in 3 phases Novice vs skilled players	Three baseline tackles were video recorded before players were shown the instructional educational video ‘‘Rugby Ready’’ demonstrating proper tackling technique. Once video was complete, players repeated the tackling exercises for an additional three trials	A Vicon MX3 Pro Motion Capture System was used to capture the motion of the tackler. Kinematics of the cervical spine, hip and knee, kinetics of the shoulder and cervical spine during a static tackle (novice vs skilled) and dynamic tackles (novice, skilled and high school). The effect of repetition to the effect of video instruction on the cervical spine Environment Controlled laboratory	Female skilled players demonstrated a larger mean knee flexion angle post-instruction (145.0 ± 3.3°) compared to pre-instruction (64.2.0 ± 23.9°). Skilled players experienced lower peak mean maximum shoulder acceleration (332.6 ± 56.6 m/s^2^ vs 459.1 ± 112.1 m/s^2^ vs, *p* < 0.01) post instruction Novice players measure larger hip angles (Novice—75.2 ± 15.7° vs. Skilled—60.5 ± 9.2°, *p* = 0.064) and post-instruction in females The educational intervention decreased cervical spine peak acceleration by a mean 51.8 m/s^2^ (SE = 46.8, *p* = 0.27) while the effect of repetition in both the pre- and post- intervention groups was shown to increase peak acceleration by a mean 11.2 m/s^2^ (SE = 13.7, *p* = 0.42) in collegiate players High school players experienced an increase in head acceleration 15.4 m/s^2^ (SE = 6.88, *p* = 0.03), while the effect of repetition showed a decrease in acceleration on average by 5.24 m/s^2^ (SE = 2.04, *p* = 0.01)
Davidow et al. (2023) [37]	24 participants -male Amateur level	Tackler	To assess the efficacy of a player-specific video-based technical feedback and instruction intervention	Non-randomised controlled	Video-based technical feedback and instruction based on technique baseline data	Video based technical feedback group (received intervention) and control group (trained on the simulator without feedback) technique compared within and between groups over time	A three sessions intervention using a baseline, intervention, and retention structure separated by at least one week using an intervention and control condition Players received specific tackle technique video and data feedback and instruction from an expert on tackling technique following a baseline session. Tackle technique was compared by shoulder dominance within and between groups Front-on one-on-one tackles were performed against a tackle dummy suspended from a pneumatic tackle contact simulator	A modified tackler technical criterion of 10 observable actions, scored by a player being awarded a zero or one point depending on whether a particular action was performed or not. These criteria divided the tackle into three phases; pre-contact, contact and post-contact. Tackle technical proficiency is presented in Arbitrary Units (AU) Environment Controlled laboratory	Only the video-based technical feedback group improved tackling technique from baseline to intervention (Feedback—6.89 [6.33–7.45] AU vs, Control—7.72 [7.35–8.10] AU *p* = 0.001 ES = 0.60 moderate) for non-dominant shoulder tackles For the retention session the feedback group scored significantly higher than the control group—for both dominant (Feedback—8.00 [7.60–8.40] AU vs. Control—7.22 [6.83–7.62 AU *p* = 0.014, ES = 0.66 moderate) and non-dominant tackles (Feedback—7.11 [7.81–8.41] AU vs. Control—7.22 [6.90–7.55] *p* = 0.004 ES = 0.96 moderate) The Video-based technical feedback group rated a decrease in mental fatigue after tackling when comparing the intervention to retention session (Feedback—25.83 [17.31–34.36] AU vs, Control—15.75 [9.10–22.40] AU *p* = 0.025 ES = 0.84 moderate)
*Rugby league*									
Speranza et al. (2016) [38]	24 participants - male semi-professional	Tackler	To examine the influence of an upper and lower body strength and power training programme on tackling ability	Repeated-measures experimental	8-week strength and power training programme	Responders vs. non-responders	8-week strength and power training programme as part of preseason training 3 times per week of 7 upper and lower body strength and power exercises	Tacker technical proficiency scoring in percentages 3RM squat and Bench press in Kg, Relative squat and bench press in Kg.kg^−1^ CMJ peak power Watts PPU peak power Watts Tackling ability measured a set of tackler technical proficiency of the players was assessed using video analysis of a standardised one-on-one tackling drill Environment Field and gym	Greater improvements in tackling ability with improved lower body strength after 8 weeks Relationship changes in tackling ability and squat 0.60 *p* < 0.01 and relative squat 0.54 *p* < 0.01, data reported as Pearson product-moment correlation coefficient, r Percentage changes in tackling ability of responder’s vs non-responders in squat 8.3 ± 10.5–1.0 ± 5.9 ES = 9.0 moderate *p* < 0.05 and relative squat 8.5 ± 7.8–1.0 ± 5.9 ES = 0.90 moderate
*Rugby union and league combined*									
Edwards et al. (2021) [40]	15 participants - male Amateur and semi-professional	Tackler	Investigate if prior instruction by expert coach to a tackler to execute different tackle types altered their tackling technique	Exploratory	Technical instruction provided by expert coach	Four tackle types divided into two categories: 1. Traditionally coached techniques - UpperNRL - LowerNRL 2. Expert coach modifications - UpperPop - MidTorso	Following a recorded static trial under guidance of an expert coach, participants were given instruction to perform a set of 10 trials of four different tackling instructions of a one-on-one front-on, torso tackle, for a total of 40 tackles. In one of two orders (i) LowerNRL, MidTorso, UpperNRL and LowerNRL, or (II) UpperNRL, UpperPop, LowerNRL and then MidTorso over one session. using tackle video and instruction on four different tackler types from an expert coach to assess if prior instruction would alter a tacklers technique	Motion capture video and Visual 3D software was used to capture and calculate the motion of the tackle participants. Kinematics and kinetics of the tackler in degrees. Velocity at peak approach to contact The kinematics of the tackle, looking at lower limb angles—ankle dorsiflexion and plantarflexion, flexion and extension of the knee, thigh, and hip. Head angles—head extension and flexion and torso angles—flexion and extension of the lumbopelvic and thoracolumbar. Resultant centre of mass velocity of both the tackler and ball-carrier across the four tackle instructions was measured Environment Controlled laboratory	Modified UpperPop instruction showed greater changes in modifying tackler technique. More head up and forward and upright posture when compared to lower torso contact instructions (*p* < 0.001) UpperPop less L5-SL flexion then UpperNRL, MidTorso and LowerNRL (*p* < 0.000) and less head flexion then UpperNRL, MidTorso and LowerNRL (*p* < 0.000) at two and one step before and at contact Approach speed for the non-dominant tackles showed increase speed for the traditional techniques. dominant (2.83 vs 2.96 m/s *P* = 0.028) with a significant difference during the MidTorso tackles (Dominant 2.31 vs Non-Dominant 2.45 m/s, *p* = 0.001)
Edwards et al. (2022) [42]	15 participants - male Amateur and semi-professional	Ball-carrier	Quantify ball-carrier’s body position when entering a front-on, one-on-one, torso tackle relative to tackler’s motion	Exploratory	Technical instruction provided by expert coach	Four tackle types divided into two categories: 1. Traditionally coached techniques - UpperNRL - LowerNRL 2. Expert coach modifications - UpperPop - MidTorso	Following a recorded static trial under guidance of an expert coach, participants were given instruction to perform a set of 10 trials of four different tackling instructions of a one-on-one front-on, torso tackle, for a total of 40 tackles. In one the order of traditional techniques LowerNRL and UpperNRL followed by the modified techniques MidTorso, or UpperPop, over one session. using tackle video and instruction on four different tackler types from an expert coach to assess if prior instruction would alter a tacklers technique	Motion capture video and Visual 3D software was used to capture and calculate the motion of the tackle participants. Kinematics and kinetics of the ball-carrier in degrees The kinematics of the tackle, looking at lower limb angles -ankle dorsiflexion and plantarflexion, flexion and extension of the knee, thigh, and hip. Head angles—head extension and flexion and torso angles—flexion and extension of the lumbopelvic and thoracolumbar. Resultant centre of mass velocity of both the tackler and ball-carrier across the four tackle instructions was measured Environment Controlled laboratory	Ball-carrier modified behaviour in response to anticipated changes in tacklers motion. Using one of two movement strategies (1) increased stability by flexing trunk (MidTorso—Dom −22.2 [−28.2–−16.2]° and non-Dom −28.6 [−34.3–−22.9]° and LowerNRL—Dom −14.8 [−19.5–−10.1]° and non-Dom—12.7 [−15.1–−10.3]° vs UpperNRL—Dom −27.0 [−34.9–−19.0] and non-Dom −28.5 [−36.0–−21.0]°, UpperPop—Dom −24.9 [−31.8–−18.0]° and nom-Dom −28.1 [−36.0–−20.3]°), knee (MidTorso—Dom 34.9 [28.3–41.5] and non-Dom 37.0 [26.9–47.1]° and LowerNRL—Dom 38.5 [31.4–45.6]° and non—Dom 39.3 [32.5–46.1], vs UpperNRL—Dom 33.7 [28.6–38.8]° and non-Dom 36.4 [29.7–43.0]°, UpperPop—Dom 32.6 [ 27.7–37.5]° and non-Dom 38.5 [30.7–46.3]°), and hips (MidTorso—Dom 547.4 [37.0–57.8]° and non-Dom 50.5 [39.9–61.1]° and LowerNRL—Dom 43.3 [35.3–51.4]° and non-Dom 42.1 [35.1–49.0]° vs UpperNRL—Dom 54.0 [42.2–65.8]° and non-Dom 51.8 [41.8–61.8]°, UpperPop—Dom 51.7 [41.6–61.8]° and nom-Dom −53.3 [42.2–64.5]°), more when entering mid or high trunk tackles: or (2) offloading the ball or performing an evasive movement strategy by positioning themselves in a more upright body position when being tackled at a low trunk height
Edwards et al. (2022) [41]	15 participants 5 both codes 2 rugby league 8 rugby union - male Amateur and semi-professional	Tackler	Investigate the influence of 4 types of successful front-on, one-on-one torso tackles on tacklers and ball-carrier’s inertial head kinematics	Exploratory	Technical instruction provided by expert coach	Four tackle types divided into two categories: 1. Smother tackle, - SNRL - SPL 2. Dominant tackle, - DTS - DNRL	Following a recorded under the guidance of an expert coach participant performed one set of traditional tackles from the NRL coaching manual were performed 5 on each shoulder. Tacklers shoulder contacted the ball-carriers upper torso (SNRL) or hip area (DNRL). When players could execute traditional tackles correctly, they could progress to expert coach instructing tacklers to perform 10 trials of two modified tackling techniques, vertical upper pop action (SPL) or redirection ball-carrier into a vertical and backward direction (DTS)	Motion capture video and Visual 3D software was used to capture and calculate the motion of the tackle participants. Kinematics of the tackler in degrees The tacklers joint angle—ankle, knee, hip, lumbopelvic, thoracolumbar, trunk-pelvis and segment angles (thigh segment, pelvis segment and trunk segment) Resultant centre of mass velocity at three points, two in the approach and one at contact Environment Controlled laboratory	Lower torso contact height (i.e. contact with hips) using the DNRL tackle instruction resulted in higher linear head acceleration (DNRL [Dom 6.2 ± 2.3] g and [ND 5.9 ± 2.1] g vs. SPL [Dom 3.9 ± 1.4] g and [ND 4.4 ± 2.0] g *p* = 0.001, SNRL [Dom 4.4 ± 1.9] g and [ND 5.1 ± 2.6] g *p* < 0.001, DTS [Dom 4.5 ± 1.6] g and ND [4.7 ± 1.7] g *p* < 0.01) and angular head acceleration (DNRL [Dom 420 ± 180] rad.s^−2^ and [ND 410 ± 167] rad.s^−2^ vs. SPL [Dom 331 ± 134] rad.s^−2^ and [ND 379 ± 205] rad.s^−2^ *p* < 0.001, SNRL [Dom 337 ± 189] rad.s^−2^ and [ND 365 ± 215] rad.s^−2^ *p* < 0.001, DTS [Dom 321 ± 136] rad.s^−2^ and ND [321 ± 110] rad.s^−2^ *p* < 0.01) for tacklers compared to all other tackle instruction types
*American Football*									
Stokes and Luiselli. (2010) [30]	1 participant - male Youth	Defence	Evaluate skill building procedures derived from functional assessment and assess performance during game conditions	Case study	Applied behavioural analysis intervention following a functional analysis of tackling skills	Session comparator of individual participant. Baseline functional assessment, intervention session and post-intervention game assessment	A baseline to intervention evaluation including a post intervention in game assessment. Baseline session involved 10 opportunities to tackle a ball-carrier with or without feedback on the ten-step tackling task. The intervention was formulated using baseline data of highest percentage of correct tackling during escape feedback. In game assessment immediately post intervention described three random tackles in game using ten-step task criteria	Ten-step tackle task analysis (%) based on skill recommendations by the American Football Coaches Association (1995) Environment Field training drill and in-game	This study showed the highest percentage of correct tackling occurred when the participant could “escape” (56.6%) with no direct verbal feedback from the coach. Compared to no attention (45%), peer attention (30%) and coach attention (25%) The postintervention assessment further supported the results by showing the player improved skills learned during the intervention scoring 75% during the ten-step tackle task analysis compared to the baseline (33%) and at the time of intervention (72%)
Harrison. (2013) [31]	3 participants - male Youth	Defence	Measure the effect of TAG and verbal instruction on tackling in high school football players	Non-randomised experimental study	Technical instruction and acoustical guidance (TAG)	Drill and session comparator of individual participants. Baseline session, shaping session and progressive speed session	A four-step tackling task over a baseline, shaping and progressive speed (walk, jog, run) stage procedure was used, participants were provided verbal instruction and shaping (TAG) to improve tackle technique. For each stage of the procedure participants were verbally instructed based on the skill task and was provide instruction for the upcoming skill and given a beep when the skill was performed correctly. Participants had to perform the skill correctly 80% of the time over 10 tackles to progress in the task	Percentage of correct tackling skills performed based on a four-skill multiple baseline shaping and progressive speed drills informed by the American Football Coaches Association (1995) Environment Training field	Participant’s tackle skill performance was low during the baseline session and increased over the shaping and progressive speed session Number of tasks required for a correct tackle was reduced over the sessions
Swartz et al. (2015) [36]	50 participants College football	Offense and defence	Test a helmetless-tackling behavioural intervention for reducing head impact exposure in National collegiate athletic association division 1 football programme	Randomised controlled trial	Helmetless tackling training	Stratified by position (offense or defence) and randomised to a helmetless intervention group and control. Randomised from position	Helmetless intervention group participated in a drill of approximately 5-min tackle drill without helmets and shoulder pads. The drill was performed twice per week during preseason (3 weeks) and once per week in season (16 weeks)	Frequency of head impacts per athlete-exposure (AE), pre, mid and end of season using a xPatch head-impact sensor Environment Field training and in-game	A 28% reduction in head impact frequency per athlete exposure (AE) at the end of the season compared to the start (9.99 ± 6.10 and 13.84 ± 7.27 *p* = 0.009) The intervention group was exposed to 30% fewer head impacts per AE compared to the control (9.99 ± 6.10 and 14.32 ± 8.45 *p* + 0.009)
Schussler et al. (2018) [32]	24 participants Youth	Defence	Examine the effect of training in a vertical, head up tackling position on head acceleration while tackling in a controlled laboratory situation	Controlled laboratory study	Reflective video and technical feedback from baseline data	One-day group and a three-day group PLA and PRA over 10 g threshold at baseline and last training group compared within groups Pretest and post-test between groups above 10 g threshold	Two groups participated in a baseline, five tackles, and intervention session, four training blocks with reflective video and technical feedback on incorrect techniques performed. During the four training sessions participants tackled following instruction on the six standard components of a heads up, vertical style tackle in the QYTS. One group performed a one-day baseline and training intervention and the second group performed two additional training sessions with a day’s rest between and a 48-h retention session with no feedback	Head impact exposure using peak head rotational (PRA) acceleration and peak linear acceleration (PLA) using a xPatch head-impact sensor, Kinematics of the participant—(Cervical extension, trunk inclination, head placement, pelvic height, shoulder extension and step length) using a Vicon motion capture system The Qualitative youth Tackling Scale (QYTS) is a visual and objective scale to instruct a vertical, head up tackling form Environment Controlled laboratory	Training in a head up and vertical style tackle reduced the number of head accelerations experienced by the tackler In the baseline session head impacts decreases from 1.2 ± 0.9 to 0.6 ± 0.7 (*p* = 0.027) after one day of training In the three-day training group Peak linear acceleration (PLA) counts over 10 g decreased from the baseline session to end of the three-day training (1.9 ± 1.6 to 0.5 ± 1.2, *p* = 0.021) After one day of training Peak rotational acceleration (PRA) decreased from 1.2 ± 0.9 to 0.5 ± 0.7 (*p* = 0.038) from baseline. In the three-day group PRA decreased from baseline to after training (1.9 ± 1.7 to 0.6 ± 1.2, *p* = 0.042) Performance in tackling using the QYTS showed an average improvement in score from baseline to the end of one day of training (1.50 ± 1.10 to 2.46 ± 1.31 *p* = 0.004)
Champagne et al. (2019) [33]	70 participants -19 wore helmet accelerometers Youth	Offense and defence	Investigate the introduction of a data-informed behavioural intervention to improve blocking and tackling techniques	Non-randomised experimental study	Baseline data-informed tackling behaviour	Between drills and within drill over time points	Video analysis from baseline session sport-specific functional assessment after the mid-season to develop a data-informed behavioural intervention designed to improve both blocking and tackling techniques while reducing head impact exposure Testing was repeated in the mid-season (internal control) without an intervention, and again in the post-season (experimental), twice weekly. A subset of participants wore in-helmet accelerometers to assess the effectiveness of the intervention in decreasing head impacts during practice	Helmet kinematics—Head impact exposure and frequencies per session using Helmet-based accelerometers and video. Head linear acceleration and rotational velocity Safety scores using predetermined tackling and blocking scoring scale. Measures number of errors, or deviations from proper technique at pre, mid and post time points Environment Indoor training turf	30% decrease in total head impact frequency per practice one month post intervention introduction Within the group tackling and blocking was shown to improve by the significant improvement in safety scores documented across all four drills (*p* < 0.0001) Average cumulative linear acceleration (g) decreased, (pre-272.19 ± 112.78 vs post-186.10 ± 80.98, *p* = 0.0037)
Swartz et al. (2019) 34]	180 participants - male Youth	Offense and defence	Investigated whether secondary school level American Football who regularly and progressively practiced tackling and blocking skills without a helmet would experience a decrease in head impacts compared to a control	Randomised controlled trial	Helmetless tackling and blocking training intervention (HuTT)	Helmetless tackle training group and control group	The HuTT intervention used a three-phase tackling and blocking progression of static, dynamic, and functional of 10 instructional drills performed without a helmet and shoulder pads compared to a control. The drill was performed four times during preseason and two times in-season for 10 min	Smart Impact Monitors measured head impact biometrics- Head impact accelerations (g’s), directional coordinates of impact location relative to head estimated centre of mass. Peak linear accelerations Environment Field training and in-game	Following the helmetless tackling intervention participants experienced 26–33% fewer game related head ImpAE (per athlete exposure) at similar time points in both seasons The intervention group had fewer head ImpAE during games compared to control at week 4 (*p* = 0.001 season 1, p = 0.0005 season 2) and 7 (*p* = 0.0001 both seasons). At week 7 during training the intervention group had fewer ImpAE compared to the control in season 1 (*p* = 0.015) Player level showed an effect as upper-level players were exposed to fewer head ImpAE and frequency decreased by 28–33% in the beginning and mid-season time points
Schussler et al. (2024) [35]	32 participants - 28 male - 4 female Youth football	Defence	Explore the effect of video and oral feedback on tackling form in youth American Football	Controlled laboratory study	Reflective video and technical oral feedback from baseline data	Four equally divided feedback model groups. Video feedback using self as model, expert as model, combined self and expert model and oral feedback Further grouped in a one-day training group and three-day training group Both groups measured at baseline, intervention, end of training, three-day group includes 48 h retention and transfer test	The video modelling and feedback intervention used video feedback using self as the model, an expert as the model, combined self and expert as the model and standardised oral feedback based on errors in QTYS performance. the oral-only feedback was the same standardised format as all video modelling groups The training consisted of a baseline test of three tackles and QYTS informed instruction. Feedback was provided in four blocks of three tackling trials by oral and video feedback per their group assignment or oral-only feedback. Real time assessment and feedback of the QTYS performance was given as oral feedback by an expert, clips of baseline tackles were used of participant based on QTYS performance and video clips of collegiate passing plays were supplied during feedback and was updated for each of the four repeated intervention blocks 3 tackles were performed for the 48-h retention and transfer task	Tackle kinematics using the Vintage; Vicon Motion System. Shoulder extension, cervical extension, trunk angle, pelvis height and step length by baseline, intervention, and end of training. Over time The Qualitative youth Tackling Scale (QYTS) is a visual and objective scale to instruct a vertical, head up tackling form Environment Controlled laboratory	The football tackle can be modified using video modelling and feedback In the one-day training group, the effect of training was seen in all movements except for trunk position. Shoulder extension progress occurred toward the goal movement over time (F_3,28_ = 8.15, *P* < .01, $\eta_{\rho}^{2}$ = 0.2), cervical extension showing that participants kept their heads up between times (F_3,28_ = 4.83, *P* = .01, $\eta_{\rho}^{2}$ = 0.3). Progress toward the goal movement between times was present for pelvis height, suggesting that the athletes lowered their centre of gravity (83% ± 5% of standing pelvis height to 77% ± 12%; F_3,28_ = 25.71, *P* < .01, $\eta_{\rho}^{2}$ = 0.7) and step length progress toward the goal movement in step length due to time (101% ± 17% of standing pelvis height to 81% ± 26%, F_3,28_ = 15.52, *P* < .01, $\eta_{\rho}^{2}$ = 0.4). Group differences were seen in pelvic height (combined—67% ± 11%, over expert—83% ± 15%; *P* < .01; d = 0.9 and oral—81% ± 6%; *P* = .01; d = 0.9) and step length (self—82% ± 26% vs oral—102% ± 30%, *P* = .02; d = 0.8 and combined—63% ± 15% vs oral—102% ± 30%; *P* < .01; d = 1.2) In the three-day group Main effects of time were identified in pelvis height (F_4,10_ = 9.02, *P* < .01, $\eta_{\rho}^{2}$ = 0.474) and step length (F_4,10_ = 9.67, *P* < .01, $\eta_{\rho}^{2}$ = 0.49). pelvis height indicated progress toward the goal movement in the percentage of standing pelvis height from baseline (83% ± 2%) to the end of training (79% ± 9%; *P* = .03; d = 0.6) and to retention (72% ± 4%; *P* < .01; d = 2.4). Retention performance in the percentage of standing pelvis height (72% ± 4%) was closer to the goal movement than transfer (85% ± 6%; *P* < .01; d = 2.6). Progress toward the goal movement was evident between times for step length from baseline (95% ± 16%) to instruction (88% ± 19%; *P* = .03; d = 0.6), to the end of training (78% ± 20%; *P* < .01; d = 0.8), and to retention (71% ± 5%; *P* < .01; d = 1.0), as well as instruction (88% ± 19%) to retention (71% ± 5%; *P* = .01; d = 0.7). Retention performance in the percentage of standing pelvis height (71% ± 5%) established greater progression toward the goal movement than transfer (99% ± 7%; *P* < .01; d = 3.7). Main effects of group were found for shoulder extension (F_3,10_ = 11.76, *P* < .01, $\eta_{\rho}^{2}$ = 0.8) and pelvis height (F_3,10_ = 5.466, *P* < .05, $\eta_{\rho}^{2}$ = 0.9). and indicated greater progress toward the goal for combined (49° ± 5°) than self (35° ± 11°; *P* < .01; d = 1.2), expert (33° ± 11°; *P* < .01; d = 1.7), and oral (27° ± 7°, *P* < .01; d = 3.2) feedback. Group differences were also noted for pelvis height in the percentage of standing pelvis height for combined (75% ± 6%) over self (81% ± 2%; *P* = .02; d = 0.7), over expert (80% ± 9%; *P* = .02; d = 0.4), and over oral (80% = 4%; P ¼ .03; d = 0.5) feedback

SE, standard error [39]; AU, arbitrary units [37]; ES, effect size [37, 38]; 3RM, three-repetition max [38]; CMJ, countermovement jump [38]; PPU, plyometric push up [38]; Dom, dominant shoulder [42]; non-Dom/ND, non-dominant shoulder [41, 42]; SNRL, tackler’s shoulder contacted the upper shoulder [41]; SPL, ball-carrier in an upward direction (pop-up) [41]; DTS, ball-carrier in a vertical backward direction [41]; DNRL, tackler’s shoulder contacted the hip area [41]; NRL, national rugby league [41]; TAG, technical instruction and acoustical guidance [31]; AE, athlete exposure [36]; PLA, peak linear acceleration [32]; PRA, peak head rotation acceleration [32]; QYTS, qualitative youth tackling scale [32, 35]; HuTT, helmetless tackling and blocking training intervention [34]; ImpAE, impact per athlete exposure [34]

### Stage 5: Collating, Summarising, and Reporting Results

This systematic scoping review presents the data in two ways. Firstly, numerically, using a flowchart (Fig. 1) displaying the selection process of the literature search and the reasons for exclusion. Secondly, studies were grouped by type of tackle collision sport and summarised for general study characteristics, study design, outcome measures, and key findings (Table 1). In addition, the sample size, participant sex, participation level, tackle role, intervention type, and comparator are reported in narrative format. The thirteen final full-text publications were assessed for quality and reported in table format (Supplementary Tables 1 and 2).

### Assessment of Methodological Quality

The final full-text publications that met the inclusion criteria (n = 13) went through a critical appraisal to assess the methodological quality (or risk bias). The quality of the full-text publications was assessed using the Joanna Briggs Institute (JBI) Critical Appraisal checklist for Quasi-experimental studies and randomised controlled trials [28]. These tools have been developed by JBI and collaborators and approved by the JBI Scientific Committee following extensive consultation [29]. For this systematic scoping review, randomised controlled trials were studies using randomisation to assign participants to one of two groups with the aim to evaluate an intervention. Quasi-experimental studies were studies evaluating interventions but which did not use randomisation to group participants i.e. used pre-existing groups, grouping by convenience or self-selection. The quality assessment was done separately by authors DD and LP. Any disagreements were resolved through discussions between the two reviewers, without the need for the involvement of a third reviewer. For the purposes of this review, studies were not eliminated based on quality.

## Results

The initial search produced a total of 5656 records—PubMed = 942, Web of Science = 1238, EBSCO Host = 2106 and Scopus = 1370. After removing the duplicates (n = 4631), 1025 titles remained for screening. Thereafter, 140 abstracts were screened for inclusion. Twenty-seven full-text articles were retrieved, and only thirteen of them met the eligibility criteria (Fig. 1).

### Sample Size, Participant Sex and Level of Participation

The field-based tackle-collision sports identified were American Football (n = 7, 54%), combined rugby union and rugby league (n = 3, 23%), rugby union (n = 2, 15%), and rugby league (n = 1, 8%). No player-level tackle training interventions were identified in Australian Rules Football, Gaelic Football, rugby sevens or Canadian Football. A total of 503 athletes participated in the thirteen studies (American Football n = 360, 72%, rugby codes n = 143, 28%). Sixty-two percent (n = 8) of these studies consisted of male participants, two studies (15%) consisted of males and females, and the remaining three studies (23%) did not report the participant sex. The majority of American Football studies were in youth football (n = 6) [30–35], with one study in college football (n = 1) [36]. In the rugby codes, participation levels included amateur (n = 1) [37], semi-professional (n = 1) [38], amateur and youth combined (n = 1) [39], and amateur and semi-professional combined (n = 3) [40–42].

### Participants’ Role in Tackle

The rugby code studies focused primarily on the tackler, with five studies measuring the outcome of the intervention on tacklers [37–41]. One study focused on the ball-carrier [42]. In American Football, studies were aimed at defensive players [30–32, 35] and both defensive and offensive players [33, 34, 36].

### Intervention Type and Description

In rugby union, the intervention types included a video-based technical feedback and instruction session based on a prior session (baseline) [37] and a tackling educational video (“*Rugby Ready*”) offered by the sport’s governing body at the time [39]. In rugby league, an 8-week strength training programme to improve tackling ability was used as an intervention [38]. In three studies from the same group using a mixed-cohort of rugby union and rugby league players, Edwards et al. (2021, 2022, 2022) used an “expert coach” to provide players with technical instruction as the technique intervention for tackling and ball-carrying into contact [40–42]. In American Football, one study used reflective verbal and video feedback [32] and another study used mixed video models and oral feedback [35]. Two studies implemented a helmetless tackle training (HuTT) programme as an intervention [34, 36]. Other studies used acoustical guidance (TAG) and verbal instruction on tackling technique [31], a data-informed behavioural intervention [33] and an applied behavioural analysis intervention following a functional tackling assessment [30].

### Comparator

Four studies compared the intervention group to a control group [32, 34, 36, 39]. Responders and non-responders to the intervention were compared in one study [38]. Three studies compared sessions or drills at baseline and different time points [30, 31, 33]. One study divided participants into a one-day and three-day training group with further groupings of four video or oral intervention models [35]. Three studies also compared the kinematics of different tackle types following the intervention using motion capture video [40–42]. One study compared the intervention group and control group over time [37].

### Outcome Measures

The outcome measures for the 13 studies included tackler [39–41] and ball-carrier kinematics based on motion capture video [42], and tackler proficiency scoring [37, 38] in rugby union and league. In American Football outcome measures included tackling task analysis [30, 31], head impact frequencies by xPatch head-impact sensor technology [32, 36], head impact frequencies using head-impact sensors (helmet or skin patches) and kinematics through motion capture systems or video [33, 34], and football tackle kinematics from a Vicon motion capture system [35].

### Study Design and Quality Assessment

Eleven studies were considered quasi-experimental design, [30–33, 35, 37–42] and two used a randomised controlled trial design [34, 36].

For quasi-experimental studies (n = 11), the average criteria score was 7 out of 9 (mode = 7, range 4–9, Supplementary Table 1) [30–33, 35, 37–42]. For the two randomised controlled studies, one study scored 10 out of 13 [36], while the other scored 11 [34] (Supplementary Table 2).

## Discussion

The purpose of this review was to determine the scope of the literature on interventions delivered directly to the player in tackle-collision sports. Given the number of studies found for each sport, it is clear that tackle training interventions delivered directly to the player are nascent. All interventions demonstrated a favorable change in the outcome measure. For example, in rugby union, video-based technical feedback and instruction improved tackling proficiency after one week [37]. In American Football, after exposure to training without a helmet, youth players experienced fewer head impacts compared to the control group [34]. Beyond the actual findings, each study also highlighted considerations for future studies aiming to test player-level tackle interventions e.g. the importance of including appropriate control conditions and retention assessments. Therefore, the collective findings and lessons from the reviewed studies provide coaches and policymakers with valuable insights into improving tackle training. while also paving the way for future research on tackle injury prevention, performance, and player development.

In most studies, the intervention was based on a form of feedback or instruction. This is not surprising, since tackling and carrying the ball into contact are contact skills, [43] and feedback and instruction are important and commonly used coaching methods [44–46]. The review highlights though that there are different channels for the delivery of this feedback and instruction. In rugby union, Kerr et al. (2018) [39] showed improvements based on an educational video. This is an important finding in rugby union, as educational videos are a key delivery channel for the sport’s governing bodies—for example, World Rugby’s “*Tackle Ready”* programme [47]. Using video feedback and instruction allows players, coaches, and practitioners to review the quality and outcomes of player movement(s), with the ability to review the video repeatedly and in slow motion. Moreover, video feedback can be combined with other forms of feedback [45]. In the reviewed studies, video feedback was combined with expert coaching instruction, teaching with acoustical guidance (TAG), technique feedback scales and criteria, and different types of reinforcement [31].

Similarly, Schussler et al. [35] (2024) demonstrated that using a mixed form of oral feedback, self-video reflection of technical errors and video of correct skill execution by an experienced athlete resulted in improvements of safe tackling technique over time in youth athletes. Considering the positive findings of combining video feedback with other forms of instruction, and the ubiquitous nature of easily capturing high-quality video, using video and instructional feedback during tackle technique training is recommended. Using a standardised technique assessment along with video feedback can also be used to inform training design and monitor players over time [14, 15, 30, 31, 33, 35, 37]. Swartz et al. [34, 36] also demonstrated how modifying the use (or non-use) of training equipment and applying non-traditional approaches to training may improve tackle behaviours in training and matches to reduce head impact exposure.

In rugby league, one study showed how improvements in muscular strength and power after an 8-week strength and power training program transferred to improvements in tackling ability [38]. This intervention followed a series of studies from the same group where they showed associations between specific physical qualities and tackling ability at different levels in rugby league [17, 48–50]. For example, Speranza et al. (2015) showed that tackling ability in semi-professional players was strongly associated with lower-body strength (3 repetitions maximum squat and relative squat), upper-body strength (3 repetition maximum bench press), and upper-body power (plyometric push-up) [38]. It has long been known that to improve tackle performance and reduce the risk of tackle injury, players need to be both technically proficient and possess tackle-specific physical attributes [51]. In other words, players in possession of tackle-specific physical attributes and high technical proficiency have a greater potential to complete more safe and effective tackles during a match. Considering studies evaluating the association between physical qualities and tackle technique in rugby league and rugby union, and the understanding of how tackle-specific physical attributes contribute to tackling safety and performance, finding only one study of player-level physical training intervention with the aim of improving tackling ability presents a promising avenue for future research in this area.

This review had a specific purpose and focused on tackle training interventions that targetted and actively involved the player. With that said, it is worth noting that educational resources with tackle training components are available to coaches [52–55]. For examples, *RugbySmart* and *BokSmart* [52, 53] in rugby union and the Heads-Up program in American Football. Most tackle-collision sport governing bodies also provide a library of tackle training resources for coaches, such as World Rugby’s *Tackle Ready* [47] or the NFL’s “*Way to play*” [56]. In 2018, a group of rugby researchers, practitioners and coaches also developed a tackle training framework and tackle load measurements to monitor tackle training [13]. To demonstrate the use of the tackle framework and skill load measurements, the authors also outline a progressive five-week tackle training programme for the pre-season, including the objectives of each session and the type of coaching instruction to complement the training goals [13]. While the efficacy and effectiveness of the progressive tackle training programme have yet to be studied, the tackle framework and training guidelines serve as a starting point to design tackle training programmes and further develop tackle training concepts [4].

Key strengths of the work to date are that the training intervention was rooted in some form of theory and/or previous research, and conducted in a team or training environment while also maintaining an experimental design. In addition to training and testing outcome measures, future studies should also consider actual match tackle technique analyses, tackle performance metrics and tackle injury risk outcomes. Also, all the work to date has been male-focused (youth and senior), and similar studies in female populations are warranted [57, 58]. Finally, the implementation of a player-level tackle training intervention may be reliant on coaching or performance context factors beyond a researcher’s expertise, and therefore it is highly recommended that researchers collaborate with experienced coaches when designing and developing tackle training interventions [21].

## Conclusions

This study reviewed the scope of the literature on interventions tested or implemented at the player level in field-based tackle-collision sports. Only thirteen studies were found—two in rugby union, one in rugby league, three using a mixed cohort of rugby league and rugby union players, and seven studies in American Football. The focus of the studies was primarily on the tackler, with the intervention incorporating a form of instruction or feedback, which was delivered through video or an expert coach. Other interventions included an 8-week strength and power training programme, designing practice sessions based on baseline data, and helmetless training in American Football. All interventions demonstrated a favourable change in the outcome measure. While the review provides coaches and policymakers with valuable insights into tackle training and highlights areas for future tackle-related research, using video feedback with other forms of instruction during tackle training and assessing tackle technique using standardised criteria are recommended. Exploring and implementing player-level tackle training interventions will further support current tackle injury prevention, performance and player development initatives.

## Supplementary Information

Supplementary file1.

## Data Availability

The datasets used and/or analysed during the current study are available from the corresponding author on reasonable request and are available in the following databases: PubMed https://pubmed.ncbi.nlm.nih.gov/, EBSCOHost https://web.p.ebscohost.com/ehost/search/selectdb?vid=0&sid=c445fd32-a09c-47d3-b047-b5a95455bb5e%40redis, Web of Science https://mjl.clarivate.com/home, Scopus https://www-scopus-com.leedsbeckett.idm.oclc.org/search/form.uri?display=basic#basic.

## Abbreviations

AFL: Australian Football League
HuTT: Helmetless tackling training
JBI: Joanna Briggs Institute
NFL: National Football League
OSF: Open Science Framework
PRISMA-ScR: The Preferred Reporting Items for Systematic Reviews and Meta-Analysis extension for Scoping Reviews
1RM: One-repetition maximum
TAG: Teaching with acoustical guidance
