# Supplementary material for: Player-Level Tackle Training Interventions in Tackle-Collision Sports: A Systematic Scoping Review
Source: Sports Med Open. 2025 Sep 10;11:103. doi: 10.1186/s40798-025-00888-9 (PMC12423000; doi:10.1186/s40798-025-00888-9)
Supplement: Supplementary file 1 — Supplementary file1. [file 40798_2025_888_MOESM1_ESM.docx]

Supplementary Material

**Player-level tackle training interventions in tackle-collision sports - a systematic scoping review**

Demi Davidow^1,2,5^, Lara Paul^1,2^, Ben Jones^1,2,5,6,7,8^, Ameer Hohlfeld^3,4^ Seipati Rasenyalo^1,2^, Kathryn Dane^9^, Isla Shill^10,11^ and Sharief Hendricks^1,5^.

^1^Division of Physiological Sciences, Department of Human Biology, Faculty of Health Sciences, University of Cape Town, Cape Town, South Africa.

^2^Health through Physical Activity, Lifestyle and Sport Research Centre, Department of Human Biology, Faculty of Health Sciences, University of Cape Town, Cape Town, South Africa.

^3^South African Medical research Council, Health Systems Research Unit, South Africa.

^4^Division of Epidemiology and Biostatistics, School of public health, Faculty of Health Sciences, University of Cape Town

^5^Carnegie Applied Rugby Research (CARR) Centre, Carnegie School of Sport, Leeds Beckett University, Leeds, UK.

^6^ School of Behavioural and Health Sciences, Faculty of Health Sciences, Australian Catholic University, Brisbane, QLD, Australia

^7^England Performance Unit, Rugby Football League, Manchester, UK.

^8^Premiership Rugby, London, UK.

^9^Discipline of Physiotherapy, School of Medicine, Trinity College Dublin, Dublin, Ireland.

^10^Sport Injury Prevention Research Centre, Faculty of Kinesiology, University of Calgary, Calgary, Alberta, Canada.

^11^Hotchkiss Brain Institute, University of Calgary, Calgary, AB, Canada

Corresponding author: Demi Davidow [demz182@gmail.com](mailto:demz182@gmail.com)

# Supplementary material 1: Electronic search strategy.

***Pubmed:***

Search key words and search: (((tackl*) AND (“relevant sport”)) AND ((keyword) OR (Keyword))

1. (((tackl*) AND (collision sport) AND ((intervention) OR (exercis*) OR (program*) OR (condition*) OR (performance) OR (injury) OR (prevention) OR (contact) OR (technique) OR (skill) OR (train*))
2. (((tackl*) AND (contact sport) AND ((intervention) OR (exercis*) OR (program*) OR (condition*) OR (performance) OR (injury) OR (prevention) OR (contact) OR (technique) OR (skill) OR (train*))
3. (((tackl*) AND (impact sport) AND ((intervention) OR (exercis*) OR (program*) OR (condition*) OR (performance) OR (injury) OR (prevention) OR (contact) OR (technique) OR (skill) OR (train*))
4. (((tackl*) AND (rugby) AND ((intervention) OR (exercis*) OR (program*) OR (condition*) OR (performance) OR (injury) OR (prevention) OR (contact) OR (technique) OR (skill) OR (train*))
5. (((tackl*) AND (rugby union) AND ((intervention) OR (exercis*) OR (program*) OR (condition*) OR (performance) OR (injury) OR (prevention) OR (contact) OR (technique) OR (skill) OR (train*))
6. (((tackl*) AND (rugby league) AND ((intervention) OR (exercis*) OR (program*) OR (condition*) OR (performance) OR (injury) OR (prevention) OR (contact) OR (technique) OR (skill) OR (train*))
7. (((tackl*) AND (rugby sevens) AND ((intervention) OR (exercis*) OR (program*) OR (condition*) OR (performance) OR (injury) OR (prevention) OR (contact) OR (technique) OR (skill) OR (train*))
8. (((tackl*) AND (Australia rules football) AND ((intervention) OR (exercis*) OR (program*) OR (condition*) OR (performance) OR (injury) OR (prevention) OR (contact) OR (technique) OR (skill) OR (train*))
9. (((tackl*) AND (Australian football league) AND ((intervention) OR (exercis*) OR (program*) OR (condition*) OR (performance) OR (injury) OR (prevention) OR (contact) OR (technique) OR (skill) OR (train*))
10. (((tackl*) AND (Canadian football) AND ((intervention) OR (exercis*) OR (program*) OR (condition*) OR (performance) OR (injury) OR (prevention) OR (contact) OR (technique) OR (skill) OR (train*))
11. (((tackl*) AND (Gaelic football) AND ((intervention) OR (exercis*) OR (program*) OR (condition*) OR (performance) OR (injury) OR (prevention) OR (contact) OR (technique) OR (skill) OR (train*))
12. (((tackl*) AND (American football) AND ((intervention) OR (exercis*) OR (program*) OR (condition*) OR (performance) OR (injury) OR (prevention) OR (contact) OR (technique) OR (skill) OR (train*))
13. (((tackl*) AND (gridiron) AND (((intervention) OR (exercis*) OR (program*) OR (condition*) OR (performance) OR (injury) OR (prevention) OR (contact) OR (technique) OR (skill) OR (train*))

***Web of science:***

Search key words and search: (((tackl*) AND (relevant sport) AND (keyword OR Keyword))

1. (tackl*) AND ((collision sport) AND (intervention OR exercis* OR program* OR condition* OR performance OR injury OR prevention OR contact OR technique OR skill OR train*))
2. ((tackl*) AND (contact sport) AND (intervention OR exercis* OR program* OR condition* OR performance OR injury OR prevention OR contact OR technique OR skill OR train*))
3. ((tackl*) AND (impact sport) AND (intervention OR exercis* OR program* OR condition* OR performance OR injury OR prevention OR contact OR technique OR skill OR train*))
4. ((tackl*) AND (rugby) AND (intervention OR exercis* OR program* OR condition* OR performance OR injury OR prevention OR contact OR technique OR skill OR train*))
5. ((tackl*) AND (rugby union) AND (intervention OR exercis* OR program* OR condition* OR performance OR injury OR prevention OR contact OR technique OR skill OR train*))
6. ((tackl*) AND (rugby league) AND (intervention OR exercis* OR program* OR condition* OR performance OR injury OR prevention OR contact OR technique OR skill OR train*))
7. ((tackl*) AND (rugby sevens) AND (intervention OR exercis* OR program* OR condition* OR performance OR injury OR prevention OR contact OR technique OR skill OR train*))
8. ((tackl*) AND (Australian rules football) AND (intervention OR exercis* OR program* OR condition* OR performance OR injury OR prevention OR contact OR technique OR skill OR train*))
9. ((tackl*) AND (Australian football league) AND (intervention OR exercis* OR program* OR condition* OR performance OR injury OR prevention OR contact OR technique OR skill OR train*))
10. ((tackl*) AND (Canadian football) AND (intervention OR exercis* OR program* OR condition* OR performance OR injury OR prevention OR contact OR technique OR skill OR train*))
11. ((tackl*) AND (Gaelic football) AND (intervention OR exercis* OR program* OR condition* OR performance OR injury OR prevention OR contact OR technique OR skill OR train*))
12. ((tackl*) AND (American football) AND (intervention OR exercis* OR program* OR condition* OR performance OR injury OR prevention OR contact OR technique OR skill OR train*))
13. ((tackl*) AND (gridiron) AND (intervention OR exercis* OR program* OR condition* OR performance OR injury OR prevention OR contact OR technique OR skill OR train*))

***EBSCOhost:***

Search key words and search: (tackl*) AND (“relevant sport”) AND (Keyword OR Keyword OR Keyword)

1. (tackl*) AND (collision sport) AND (intervention) OR (exercis*) OR (program*) OR (condition*) OR (performance) OR (injury) OR (prevention) OR (contact) OR (technique) OR (skill) OR (train*))
2. (tackl*) AND (contact sport) AND (intervention) OR (exercis*) OR (program*) OR (condition*) OR (performance) OR (injury) OR (prevention) OR (contact) OR (technique) OR (skill) OR (train*))
3. (tackl*) AND (impact sport) AND (intervention) OR (exercis*) OR (program*) OR (condition*) OR (performance) OR (injury) OR (prevention) OR (contact) OR (technique) OR (skill) OR (train*))
4. (tackl*) AND (rugby) AND (intervention) OR (exercis*) OR (program*) OR (condition*) OR (performance) OR (injury) OR (prevention) OR (contact) OR (technique) OR (skill) OR (train*))
5. (tackl*) AND (rugby union) AND (intervention) OR (exercis*) OR (program*) OR (condition*) OR (performance) OR (injury) OR (prevention) OR (contact) OR (technique) OR (skill) OR (train*))
6. (tackl*) AND (rugby league) AND (intervention) OR (exercis*) OR (program*) OR (condition*) OR (performance) OR (injury) OR (prevention) OR (contact) OR (technique) OR (skill) OR (train*))
7. (tackl*) AND (rugby sevens) AND (intervention) OR (exercis*) OR (program*) OR (condition*) OR (performance) OR (injury) OR (prevention) OR (contact) OR (technique) OR (skill) OR (train*))
8. (tackl*) AND (Australian rules football) AND (intervention) OR (exercis*) OR (program*) OR (condition*) OR (performance) OR (injury) OR (prevention) OR (contact) OR (technique) OR (skill) OR (train*))
9. (tackl*) AND (Australian football league) AND (intervention) OR (exercis*) OR (program*) OR (condition*) OR (performance) OR (injury) OR (prevention) OR (contact) OR (technique) OR (skill) OR (train*))
10. (tackl*) AND (Canadian football) AND (intervention) OR (exercis*) OR (program*) OR (condition*) OR (performance) OR (injury) OR (prevention) OR (contact) OR (technique) OR (skill) OR (train*))
11. (tackl*) AND (Gaelic football) AND (intervention) OR (exercis*) OR (program*) OR (condition*) OR (performance) OR (injury) OR (prevention) OR (contact) OR (technique) OR (skill) OR (train*))
12. (tackl*) AND (American football) AND (intervention) OR (exercis*) OR (program*) OR (condition*) OR (performance) OR (injury) OR (prevention) OR (contact) OR (technique) OR (skill) OR (train*))
13. (tackl*) AND (gridiron) AND (intervention) OR (exercis*) OR (program*) OR (condition*) OR (performance) OR (injury) OR (prevention) OR (contact) OR (technique) OR (skill) OR (train*))

***Scopus:***

Search key words and search: ( tackl* ) AND ( “relevant sport” ) AND ( keyword ) OR ( keyword ) )

1. ( tackl* ) AND ( collision AND sport ) AND ( intervention ) OR ( exercis* ) OR ( program* ) OR ( condition* ) OR ( performance ) OR ( injury ) OR ( prevention ) OR ( contact ) OR ( technique ) OR ( skill ) OR ( train* ) )
2. ( tackl* ) AND (contact AND sport) AND ( intervention ) OR ( exercis* ) OR ( program* ) OR ( condition* ) OR ( performance ) OR ( injury ) OR ( prevention ) OR ( contact ) OR ( technique ) OR ( skill ) OR ( train* ) )
3. ( tackl* ) AND (impact AND sport) AND ( intervention ) OR ( exercis* ) OR ( program* ) OR ( condition* ) OR ( performance ) OR ( injury ) OR ( prevention ) OR ( contact ) OR ( technique ) OR ( skill ) OR ( train* ) )
4. ( tackl* ) AND ( rugby ) AND ( intervention ) OR ( exercis* ) OR ( program* ) OR ( condition* ) OR ( performance ) OR ( injury ) OR ( prevention ) OR ( contact ) OR ( technique ) OR ( skill ) OR ( train* ) )
5. ( tackl* ) AND ( rugby AND union ) AND ( intervention ) OR ( exercis* ) OR ( program* ) OR ( condition* ) OR ( performance ) OR ( injury ) OR ( prevention ) OR ( contact ) OR ( technique ) OR ( skill ) OR ( train* ) )
6. ( tackl* ) AND ( rugby AND league ) AND ( intervention ) OR ( exercis* ) OR ( program* ) OR ( condition* ) OR ( performance ) OR ( injury ) OR ( prevention ) OR ( contact ) OR ( technique ) OR ( skill ) OR ( train* ) )
7. ( tackl* ) AND ( rugby AND sevens ) AND ( intervention ) OR ( exercis* ) OR ( program* ) OR ( condition* ) OR ( performance ) OR ( injury ) OR ( prevention ) OR ( contact ) OR ( technique ) OR ( skill ) OR ( train* ) )
8. ( tackl* ) AND ( Australian AND rules AND football ) AND ( intervention ) OR ( exercis* ) OR ( program* ) OR ( condition* ) OR ( performance ) OR ( injury ) OR ( prevention ) OR ( contact ) OR ( technique ) OR ( skill ) OR ( train* ) )
9. ( tackl* ) AND ( Australian AND football AND league ) AND ( intervention ) OR ( exercis* ) OR ( program* ) OR ( condition* ) OR ( performance ) OR ( injury ) OR ( prevention ) OR ( contact ) OR ( technique ) OR ( skill ) OR ( train* ) )
10. ( tackl* ) AND ( Canadian AND football ) AND ( intervention ) OR ( exercis* ) OR ( program* ) OR ( condition* ) OR ( performance ) OR ( injury ) OR ( prevention ) OR ( contact ) OR ( technique ) OR ( skill ) OR ( train* ) )
11. ( tackl* ) AND ( Gaelic AND football ) AND ( intervention ) OR ( exercis* ) OR ( program* ) OR ( condition* ) OR ( performance ) OR ( injury ) OR ( prevention ) OR ( contact ) OR ( technique ) OR ( skill ) OR ( train* ) )
12. ( tackl* ) AND ( American AND football ) AND ( intervention ) OR ( exercis* ) OR ( program* ) OR ( condition* ) OR ( performance ) OR ( injury ) OR ( prevention ) OR ( contact ) OR ( technique ) OR ( skill ) OR ( train* ) )
13. ( tackl* ) AND ( gridiron ) AND ( intervention ) OR ( exercis* ) OR ( program* ) OR ( condition* ) OR ( performance ) OR ( injury ) OR ( prevention ) OR ( contact ) OR ( technique ) OR ( skill ) OR ( train* ) )

All databases were last searched on 28 May 2024.

# Supplementary material 2: JBI Critical appraisal tables

Supplementary Table 1 – JBI quality assessment for Quasi experimental studies (non-randomised)”

|  |  | | | | | | | | | | | | | | |
| --- | --- | --- | --- | --- | --- | --- | --- | --- | --- | --- | --- | --- | --- | --- | --- |
| Questions | |  | |  | | **Author(s) (year)** | | | | | | | | | |
|  | | Kerr et al. (2018) [39] | Schussler et al. (2018) [32] | | Schussler et al. (2024) [35] | | Speranza et al. (2016) [38] | Stokes and Luiselli. (2010) [30] | Champagne et al. (2019) [33] | Davidow et al. (2023) [37] | Edwards et al. (2021) [40] | Edwards et al. (2022) [41] | Edwards et al. (2022) [42] | Harrison. (2013) [31] |  |
| 1. Is it clear in the study what is the ‘cause’ and what is the ‘effect; (i.e., there is no confusion about which variable comes first)? | | Yes | Yes | | Yes | | Yes | Yes | Yes | Yes | Yes | Yes | Yes | Yes |  |
| 1. Were the participants included in any comparisons similar? | | Yes | Yes | | Yes | | Yes | Yes | Yes | Yes | Yes | Yes | Yes | Yes |  |
| 1. Were the participants included in any comparisons receiving similar treatment/care, other than the exposure or intervention of interest? | | No | Yes | | Yes | | Yes | Yes | No | Yes | No | No | No | No |  |
| 1. Was there a control group? | | No | No | | Yes | | No | No | Yes | Yes | No | No | No | No |  |
| 1. Were there multiple measurements of the outcomes both pre and post the intervention/exposure? | | Yes | Yes | | Yes | | Yes | Yes | Yes | Yes | No | Yes | Yes | Yes |  |
| 1. Was the follow up complete and if not, were differences between groups in terms of their follow up adequately described and analysed? | | Yes | Yes | | Yes | | Yes | Yes | Yes | Yes | No | No | No | No |  |
| 1. Were the outcomes of participants included in comparisons measured in the same way? | | Yes | Yes | | Yes | | Yes | Yes | Yes | Yes | Yes | Yes | Yes | Yes |  |
| 1. Were the outcomes measured in a reliable way? | | Yes | No | | Yes | | Yes | Yes | No | Yes | No | No | Yes | Yes |  |
| 1. Was appropriate statistical analysis used? | | Yes | Yes | | Yes | | Yes | No | Yes | Yes | Yes | Yes | Yes | Yes |  |
| Total (/9) | | **7** | **7** | | **9** | | **8** | **7** | **7** | **9** | **4** | **5** | **7** | **6** |  |
|  | | **77.8%** | **77.8%** | | **100%** | | **88.8%** | **77.8%** | **77.8%** | **100%** | **44.4%** | **55.6%** | **77.8%** | **66.7%** |  |

Supplementary Table 2. JBI Quality assessment for Randomised Control Trials.

| Questions | Author(s) (year) | |
| --- | --- | --- |
|  | Swartz et al. (2015) [36] | Swartz et al. (2019) [34] |
| 1. Was true randomisation used for assignment of participants to treatment groups? | Unclear | Yes |
| 1. Was allocation to treatment groups concealed? | Yes | Yes |
| 1. Were treatment groups similar at the baseline? | Yes | Yes |
| 1. Were participants blind to treatment assignment? | Yes | Yes |
| 1. Were those delivering treatment blind to treatment assignment? | Unclear | No |
| 1. Were outcomes assessors blind to treatment assignment? | Yes | Yes |
| 1. Were the treatment groups treated identically other than the intervention of interest? | Unclear | Unclear |
| 1. Was follow up complete and if not, were differences between groups in terms of follow up adequately described and analysed? | Yes | Yes |
| 1. Were participants analysed in the groups to which they were randomised? | Yes | Yes |
| 1. Were outcomes measured in the same way for the treatment groups? | Yes | Yes |
| 1. Were outcomes measured in a reliable way? | Yes | Yes |
| 1. Was appropriate statistical analysis used? | Yes | Yes |
| 1. Was the trial design appropriate, and any deviations from the standard RTC design (Individual randomisation. Parallel groups) accounted for in the conduct and analysis of the trial? | Yes | Yes |
| Total (/13) | **10** | **11** |
|  | **76.9%** | **86.6%** |
